# SIRT5-related lysine demalonylation of GSTP1 contributes to cardiomyocyte pyroptosis suppression in diabetic cardiomyopathy

**DOI:** 10.7150/ijbs.83306

**Published:** 2024-01-01

**Authors:** Can Wei, Meixin Shi, Shiyun Dong, Zhitao Li, Bingbing Zhao, Dan Liu, Guopeng Li, Jie Cen, Ligen Yu, Xiao Liang, Lili Shi

**Affiliations:** 1 Department of Pathophysiology, Harbin Medical University, Harbin 150086, Heilongjiang, P.R. China.; 2 Department of Cadre ward, The Fourth Affiliated Hospital of Harbin Medical University, Harbin, 150081, Heilongjiang, P.R. China.; 3 Department of Cardiology, The First Affiliated Hospital of Harbin Medical University, Harbin, 150081, Heilongjiang, P.R. China.; 4 Department of Cadre Ward, The First Affiliated Hospital of Harbin Medical University, Harbin, 150081, Heilongjiang, P.R. China.

**Keywords:** Diabetic cardiomyopathy, SIRT5, GSTP1, SPI1, Lysine malonylation

## Abstract

Sirtuin 5 (SIRT5), localized in the mitochondria, has been identified as a protein desuccinylase and demalonylase in the mitochondria since the depletion of SIRT5 boosted the global succinylation and malonylation of mitochondrial proteins. We investigated the role of SIRT5 in diabetic cardiomyopathy (DCM) and identified the mechanism regarding lysine demalonylation in this process. Wild-type and SIRT5 knockout mice were induced with DCM, and primary cardiomyocytes and cardiac fibroblasts extracted from wild-type and SIRT5 knockout mice were subjected to high glucose (HG). SIRT5 deficiency exacerbated myocardial injury in DCM mice, aggravated HG-induced oxidative stress and mitochondrial dysfunction in cardiomyocytes, and intensified cardiomyocyte senescence, pyroptosis, and DNA damage. DCM-induced SIRT5 loss diminished glutathione S-transferase P (GSTP1) protein stability, represented by significantly increased lysine malonylation (Mal-Lys) modification of GSTP1. SIRT5 overexpression alleviated DCM-related myocardial injury, which was reversed by GSTP1 knockdown. Reduced SIRT5 transcription in DCM resulted from the downregulation of SPI1. SPI1 promoted the transcription of SIRT5, thereby ameliorating DCM-associated myocardial injury. However, SIRT5 deletion resulted in a significant reversal of the protective effect of SPI1. These observations suggest that SPI1 activates SIRT5 transcriptionally to mediate GSTP1 Mal-Lys modification and protein stability, thus ameliorating DCM-associated myocardial injury.

## Introduction

Diabetic cardiomyopathy (DCM) is a diabetes mellitus-induced pathophysiological condition that can lead to heart failure 1. DCM is initially characterized by myocardial fibrosis, dysfunctional remodeling, and diastolic dysfunction, later by systolic dysfunction, and eventually by clinical heart failure 2. Mitochondrial dysfunction, promotions in oxidative stress, increases in advanced glycation end products and collagen-based cardiomyocyte and extracellular matrix (ECM) stiffness, reduced mitochondrial and cardiomyocyte calcium handling, and inflammation have been implicated in the progression of DCM 3, 4. Pyroptosis, a type of programmed cell death, is activated by bacteria, pathogens, or their endotoxins, accompanied by the activation of the caspase family, inflammasome activation, and the release of cell contents and inflammatory mediators, thus playing an important role in the pathogenesis of cardiovascular diseases, including DCM 5, 6. Intriguingly, hyperglycemia-induced reactive oxygen species (ROS) overproduction may promote the activation of nucleotide-binding oligomerization domain-like receptor (NLR) pyrin domain containing 3 (NLRP3) inflammasome which regulates the death of cardiomyocytes and activation of fibroblasts in DCM 7, 8. Therefore, we postulated in this study that oxidative stress stimulated in DCM induced the activation of NLRP3 and the ensuing pyroptosis, thereby governing the death of cardiomyocytes and activation of fibroblasts.

Protein lysine malonylation (Mal-Lys), a newly identified post-translational modification, has been revealed to be implicated in type 2 diabetic mice and enriched in metabolic-associated proteins 9. In 2011, lysine (K) malonylated substrates were first detected by mass spectrometry and protein sequence database searching, and Sirtuin 5 (SIRT5), localized in the mitochondria, can catalyze lysine demalonylation and lysine desuccinylation modification both *in vitro* and *in vivo* 10. SIRT5 knockout (KO) mice show global protein hypersuccinylation and hypermalonylation, indicating that SIRT5 is the major protein desuccinylase and demalonylase in mitochondria 11. For instance, SIRT5 has been recently reported to desuccinylase the K108 site of optineurin, thus protecting retinal ganglion cells from high glucose (HG)-induced autophagic flux blockade in the diabetic retina 12. However, there is little information regarding the regulatory role of SIRT5 in lysine demalonylation in DCM.

Here, by integrating a published report 13, KEGG enrichment analysis, and subcellular localization analysis using a bioinformatics website, we identified glutathione S-transferase P (GSTP1) as a possible substrate for SIRT5-catalyzed lysine demalonylation. GSTP1 represents the most predominant mammalian isoenzyme of the glutathione S-transferase family, which has an imperative role in detoxification and antioxidant defense 14. Consequently, we hypothesized that lysine demalonylation of GSTP1 governed by SIRT5 affects the development of DCM via oxidative stress and pyroptosis.

## Materials and Methods

### Establishment of animal models

CRISPR/Cas9-based 6-week-old male SIRT5 KO mice (C57BL/6N-Sirt5^em1Cya^, Product No. S-KO-12694) were purchased from Cyagen (Suzhou, Jiangsu, China). Wild-type (WT) 6-week-old male C57BL/6N mice were provided by the Animal Center of Harbin Medical University (Harbin, China). The mice were treated following the Guide for the Care and Use of Laboratory Animals as adopted and promulgated by the US NIH. All treatment protocols were approved by the Institutional Animal Care and Use Committee at the Experimental Animal Center of Harbin Medical University (Harbin, Heilongjiang, China). The mice were placed in a specific-pathogen-free environment with access to feed and water with alternate 12-hour light and dark cycles at 24 ± 1°C with 60 ± 10% relative humidity.

The DCM mice were generated by intraperitoneal injection of streptozotocin (STZ, Sigma-Aldrich Chemical Company, St Louis, MO, USA; 55 mg/kg per day) dissolved in citrate buffer (pH = 4.5) for 5 consecutive days as previously reported 15. Two weeks after STZ injections, mice with fasting blood glucose (FBG) ≥ 16.7 mM were selected as successful DCM mouse models for subsequent experiments (defined as week 0). Ten experimental mice were guaranteed in each group. A total of 10 KO mice and 90 WT mice were used for the following assays. WT mice injected with citrate buffer alone (n = 10) served as controls (Control group). The mice were subsequently monitored for FBG and body weight for 24 weeks.

Adeno-associated virus (AAV) packaged with short hairpin RNA (shRNA) targeting SIRT5 (AAV-shSIRT5), AAV-shGSTP1, AAV-SPI1, AAV-SIRT5, and control viruses (AAV-NC or AAV-shNC) with a mouse α-cardiac myosin heavy chain promoter were acquired from VectorBuilder (Guangzhou, Guangdong, China) with a viral titer of 2 × 10^11^ GC/mL. The mice were injected *in situ* at week 0 and week 12, and five sites were selected in each mouse to inject a total volume of 10 μL of AAV.

### Echocardiographic function

At 24 weeks, the mice were tested for cardiac function using a Vevo 1100 ultrasound machine (VisualSonics, Toronto, Canada). Animals were anesthetized under 1% sodium pentobarbital (*ip*, 50 mg/kg), and the heart was imaged in a 2-dimensional parasternal short-axis view. M-mode echocardiograms were recorded at the level of the papillary muscle in the middle of the ventricle. Ejection fraction (EF) and shortening fraction (FS) were measured on the M-mode trajectory and calculated by the machine as isovolumic relaxation time (IVRT). Hemodynamic data were collected for analysis.

### Histochemical staining

After echocardiographic analysis, the mice were fasted overnight and euthanized by pentobarbital (150 mg/kg, *ip*) overdose anesthesia, and tissues were collected. The hearts of mice were carefully dissected from the surrounding tissues and cut into 2 parts. One part of the tissue sample was fixed in 4% paraformaldehyde, embedded in paraffin, and dehydrated in gradient sucrose for hematoxylin-eosin (HE), Mason's trichrome staining, and immunohistochemistry. The remaining part of the tissue was stored at -80°C for the following experiments.

Myocardial tissues were fixed in 4% paraformaldehyde, paraffin-embedded, and sectioned (3 μm). Tissue sections were stained with HE Staining Kit (C0105S, Beyotime Biotechnology Co., Ltd., Shanghai, China) and Masson Trichrome Staining Kit (BP-DL021, Nanjing SenBeiJia Biological Technology Co., Ltd., Nanjing, Jiangsu, China), respectively. Immunohistochemistry was conducted using antibodies against GSTP1 (1:200, 15902-1-AP, ProteinTech Group, Chicago, IL, USA) and SPI1 (1:100, #2258, Cell Signaling Technologies, Beverly, MA, USA). Myocardial histomorphology, extracellular collagen deposition, and positive immunohistochemical staining (brownish) were observed under the microscope.

### Primary cell culture

Cardiomyocytes were isolated from WT mice (n = 5) and KO mice (n = 5) as described previously 16 and cultured in minimal essential medium (Gibco, Carlsbad, CA, USA) supplemented with 10% FBS and 1% penicillin/streptomycin at 37°C and 5% CO_2_.

Cardiac fibroblasts were isolated from WT mice (n = 5) as described previously 17 and cultured in DMEM (Gibco) containing 10% FBS and 1% penicillin/streptomycin for co-culture with the conditioned medium (CM) of WT or KO cardiomyocytes.

### Cellular immunofluorescence

The isolated primary cells were fixed by 4% paraformaldehyde and subsequently permeabilized by TritonX-100. The primary cardiomyocytes or primary cardiac fibroblasts were incubated with α-Sarcometric actin (1:500, ab137346, Abcam, Cambridge, UK) or Vimentin (1:500, ab8978, Abcam) at 4°C overnight, respectively. Appropriate fluorescent secondary antibodies were subsequently added to detect the protein signal, and DAPI was used for the nuclei counter-staining. The staining was observed by confocal fluorescence microscopy.

### Plasmid construction, transfection, and culture

The DNA overexpression plasmids oe-SPI1 and oe-SIRT5 based on the pEXP-RB-Mam vector were purchased from Guangzhou RiboBio Co., Ltd. (Guangzhou, Guangdong, China). The shRNAs based on mammalian shRNA interfering with the piggyBac vector (Loop sequence: CTCGAG) were purchased from VectorBuilder. The DNA plasmids or shRNAs were transfected into primary cardiomyocytes using Lipofectamine 3000. The shRNA sequences were as follows: sh-GSTP1 1#: 5'-GCCCAGATGGATATGGTGAATCTCGAGATTCACCATATCCATCTGGGC-3'; sh-GSTP1 2#: 5'-CACCAACTATGAGAATGGTAACTCGAGTTACCATTCTCATAGTTGGTG-3'; sh-GSTP1 3#: 5'-CGGCAAATATGTCACCCTCATCTCGAGATGAGGGTGACATATTTGCCG-3'; sh-SIRT5 1#: 5'-CGACAGATTCAGGTTTCATTTCTCGAGAAATGAAACCTGAATCTGTCG-3'; sh-SIRT5 2#: 5'-CCAGTTGTGTTGTAGACGAAACTCGAGTTTCGTCTACAACACAACTGG-3'; sh-SIRT5 3#: 5'-AGTTCAAATATGGCAGACTTTCTCGAGAAAGTCTGCCATATTTGAACT-3'.

Using the same protocol as our previous report 18, the cells were cultured with DMEM (5.5 mM glucose, 10% FBS), transfected, and induced with 40.0 mM glucose (the HG group), and cells treated with 5.5 mM glucose served as the control (NG). Then, all the cardiomyocytes were incubated for 48 h. Following the manufacturer's protocol, the cells in the HG or NG groups were treated with the cellular pyroptosis inhibitor INF200 (HY-155517, MedChemExpress, Monmouth Junction, NJ, USA) at a concentration of 10 μM for 48 h. Cells with an equal dose of dimethylsulfoxide (DMSO) were added as a control.

### Microscopy

After the indicated cell treatments, the cells were observed under a light microscope, and the number of pyroptotic cells (typical characteristics: swollen and expanded cells with loss of cell membrane integrity and containing bubble-like protrusions) were counted. Three fields of view were randomly selected, and the number of pyroptotic cells was counted in 50 cells under each field of view. The average number of pyroptotic cells was calculated.

### RT-qPCR

Total RNA was extracted from cells by TRIzol (Beyotime). A StarScript II RT Kit (GenStar BioSolutions, Beijing, China) was used for first-strand cDNA synthesis, followed by qPCR reactions on ABI prism7000 by RealStar Fast SYBR qPCR Mix (GenStar). Gene expression was quantified relative to the geometric mean of housekeeping gene (GAPDH) expression using the 2^-ΔΔCT^ method. The sequence information of primers used in RT-qPCR analysis is shown in Table 1.

### Western blot

Minute Mitochondrial Isolation Kit for Mammalian Cells and Tissues (MP-007, Invent Biotechnologies, Inc., Beijing, China) was used for the isolation of mitochondrial fractions from cells or tissues.

Whole-cell fractions, mitochondrial fractions, or total proteins from tissues were extracted using radioimmunoprecipitation assay lysis buffer (Beyotime). After protein concentration determination using Pierce BCA Protein Quantification Kit (Thermo Fisher), appropriate protein samples were separated using sodium dodecyl sulfate-polyacrylamide gel electrophoresis. The resolved proteins were transferred to a polyvinylidene difluoride membrane. The membrane was blocked with 5% skim milk for 2 hours at room temperature and probed with the following antibodies: β-Tubulin (1:500, ab6046, Abcam), SIRT5 (1:1000, ab259967, Abcam), SIRT3 (1:1000, ab246522, Abcam), SIRT4 (1:1000, #24662, Cell Signaling Technologies), SIRT1 (1:1000, ab189494, Abcam), COX IV (1:2000, ABL1060, Abbkine Scientific Co., Ltd., Wuhan, Hubei, China), Cleaved-caspase1 (1:1000, 89332, Cell Signaling Technologies), NLRP3 (1:1000, ab263899, Abcam), GSDMD-N (1:1000, 50928, Cell Signaling Technologies), Collagen Ⅰ (1:1000, ab255809, Abcam), Collagen Ⅲ (1:1000, PA5-34787, Thermo Fisher Scientific), GSTP1 (1:1000, 15902-1-AP, ProteinTech), SPI1 (1:1000, #2258, Cell Signaling Technologies), and Malonyl-Lysine [Mal-Lys] MultiMab™ Rabbit mAb mix (1:1000, #14942, Cell Signaling Technologies) overnight at 4°C. Then, the membranes were subsequently incubated with secondary antibodies at room temperature for 2 h. Protein signals were detected using the ECL Substrate Kit (Abcam), and protein bands were quantified by ImageJ software. β-Tubulin served as an internal reference for whole cell fractions or tissue proteins, and COX IV as an internal reference for mitochondrial proteins. Protein levels were calculated from the ratio of the corresponding protein/the internal reference.

### Co-immunoprecipitation (Co-IP)

The Pierce™ Co-IP Kit (Thermo Fisher) was used according to the manufacturer's instructions. First, different antibodies were incubated with protein A/G magnetic beads. Cell lysates from cardiomyocytes were then collected and incubated with antibody-coupled beads. The proteins interacting with the magnetic beads were washed and denatured, and the expression of the associated proteins was detected using Western blot.

### Cell counting kit-8 (CCK-8)

CCK-8 (Beyotime) was used to detect cardiomyocyte viability or cardiac fibroblast proliferation. The cells were seeded into 96-well plates at 3000 cells/well and treated or cultured accordingly, followed by the addition of 10 μL CCK-8 solution. After 2 h, the OD value at 450 nm was read to measure the viability or proliferation of the cells.

### Flow cytometry

An annexin-fluorescein isothiocyanate (FITC)/propidium iodide (PI) Apoptosis Assay Kit (Beijing Solarbio Life Sciences Co., Ltd., Beijing, China) was used to analyze the apoptosis of cardiomyocytes. Briefly, the treated cardiomyocytes were resuspended with 1× binding buffer to adjust the concentration of 5 × 10^6^ cells/mL. After that, the cell suspension (100 µL) was incubated with 5 µL Annexin V/FITC for 5 min at room temperature in the dark. With the addition of 5 µL PI and 400 µL phosphate-buffered saline, the cells were immediately loaded for flow cytometry.

### Terminal deoxynucleotidyl transferase dUTP nick end labeling (TUNEL) assay

The one-step TUNEL Apoptosis Detection Kit (Green Fluorescence, Beyotime) was used to analyze DNA damage in cardiomyocytes. Treated cardiomyocytes were fixed with 4% paraformaldehyde for 30 min and permeabilized by 0.3% Triton X-100 for 5 min at room temperature. Subsequently, the cells were incubated with TUNEL assay solution for 60 min at 37°C, followed by nuclear labeling with DAPI for 10 min at room temperature (both in the dark). After sealing with an anti-fluorescence quenching sealing solution (Beyotime), the slices were observed under a fluorescence microscope, and the percentage of TUNEL-positive cells was calculated.

### Enzyme-linked immunosorbent assay (ELISA)

The Mouse IL-6 ELISA Kit (ab222503, Abcam), Mouse TNF-α ELISA Kit (NBP2-31085, Novus Biological Inc., Littleton, CO, USA), Mouse IL-1β ELISA Kit (ab197742, Abcam), and Mouse IL-18 ELISA Kit (ab216165, Abcam) were used to detect the concentration of pro-inflammatory factors in the supernatant of cardiomyocytes as per the manufacturer's protocol. Mouse advanced glycation end-products (AGEs) ELISA Kit (Ek-M20923, Shanghai EK-Bioscience Biotechnology Co., Ltd., Shanghai, China) was used to analyze the concentration of AGEs in mouse myocardial tissues.

### Oxidative stress indicator tests

The protein Carbonyl Content Assay Kit (ab126287, Abcam), PicoProbe™ Reduced Glutathione (GSH) Assay Kit (K740, BioVision, Inc., Exton, PA, USA), Superoxide Dismutase (SOD) Activity Colorimetric Assay Kit (K335, Biovision) were applied for the determination of oxidative stress-related indicators in cardiomyocyte lysates. MitoSOX™ Red (M36008, Invitrogen Inc., Carlsbad, CA, USA) staining was performed in live cells to analyze mtROS production. Intracellular ROS levels were assessed by a ROS Assay kit (Elabscience, Wuhan, Hubei, China).

### Cell senescence detection

Immunosenescence has been identified as a hallmark of prolonged low-grade systemic inflammation and can be a cause as well as a consequence of T2D 19. A Cellular Senescence β-Galactosidase Staining Kit (C0602, Beyotime) was used to analyze the senescence of cells. For cardiomyocytes cultured in 6-well plates, 1 mL β-galactosidase staining fixative was added for a 15-minutes fixation at room temperature. Subsequently, 1 mL of staining working solution was added to each well and incubated overnight at 37°C. The number of positive cells was observed under an ordinary light microscope and counted.

### Seahorse assay

Oxygen consumption rate (OCR) analysis was performed on a Seahorse XF Pro Analyzer (Agilent Technologies, Santa Clara, CA, USA) using the Seahorse XF Cell Mito Stress Test Kit (Agilent Technologies) to detect mitochondrial respiratory function in cardiomyocytes. The cells were washed with XF assay medium (unbuffered DMEM + 10 mM glucose + 2 mM L-glutamine + 1 mM sodium pyruvate) 1 h before the assay and incubated in 500 μL basal medium in a CO_2_-free incubator at 37°C. OCR was analyzed by sequential auto-injection of mitochondrial inhibitors, including 2.5 μM oligomycin, 2 μM FCCP, 0.5 μM rotenone, and 0.5 μM antimycin A. OCR (pmol/min) was measured according to the manufacturer's instructions, and the results were used to calculate basal respiration, maximal respiration, adenosine triphosphate (ATP) production (normalized to 1 μg protein). Basal respiration = mean OCR (10 min + 20 min + 30 min) - mean OCR (100 min + 110 min + 120 min). ATP production = mean OCR (10 min + 20 min + 30 min) - mean OCR (40 min + 50 min + 60 min). Maximal respiration = mean OCR (70 min + 80 min + 90 min) - mean OCR (100 min + 110 min + 120 min).

### Protein stability assay

The transfected cardiomyocytes were treated with CHX (50 μM) for the indicated time intervals (0, 15, 30, 60, and 120 min). The cells were harvested to isolate mitochondrial proteins, and GSTP1 expression was measured by Western blot (internal reference: COX IV). The quantified GSTP1 protein was normalized according to the protein expression at 0 minute to calculate the protein degradation rate.

### ChIP

The binding of SPI1 to the SIRT5 promoter was analyzed using Pierce™ Agarose ChIP Kit (Thermo Fisher). Protein-DNA complexes were stabilized by 4% paraformaldehyde. After ultrasonic fragmentation, protein-DNA complexes were immunoprecipitated by primary antibody to SPI1 (1:100, #2258, Cell Signaling Technologies) or rabbit IgG (1:100, #66362, Cell Signaling Technologies). The cross-linked complexes were extracted and solubilized, and the enrichment levels of the SIRT5 promoter were analyzed by qPCR.

### Luciferase assay

The promoter sequence of SIRT5 (chr13: 43,365,384-43,365,730) was obtained from the UCSC Genome Browser (https://genome.ucsc.edu/) and inserted into pGL3-Basic Luciferase Reporter Vectors (Promega Corporation, Madison, WI, USA) to construct the wild type (wt) SIRT5 promoter reporter plasmid. In addition, mutant (mut) SIRT5 promoter reporter plasmids containing mutated SIRT5 promoter sequences were constructed by point mutation. The above plasmids were transfected into cardiomyocytes by Lipofectamin3000 alone (Blank group) or with oe-NC and oe-SPI1, respectively, followed by HG treatment. After 48 h, luciferase activity was detected by the Dual-Luciferase® Reporter Assay System (Promega).

### Statistical analysis

Continuous data were expressed as mean ± SD. After testing for normality using the Shapiro-Wilk test, continuous data were compared by unpaired *t*-test or one-way/two-way analysis of variance (ANOVA). Post hoc Tukey's or Sidak's tests were performed where significant interactions were observed in ANOVAs. GraphPad Prism 8.0 (GraphPad, San Diego, CA, USA) was used for statistical analysis, and statistical significance was set at *p* < 0.05.

## Results

### SIRT5 deficiency exacerbates cardiac injury in DCM mice

**Figure 1:**
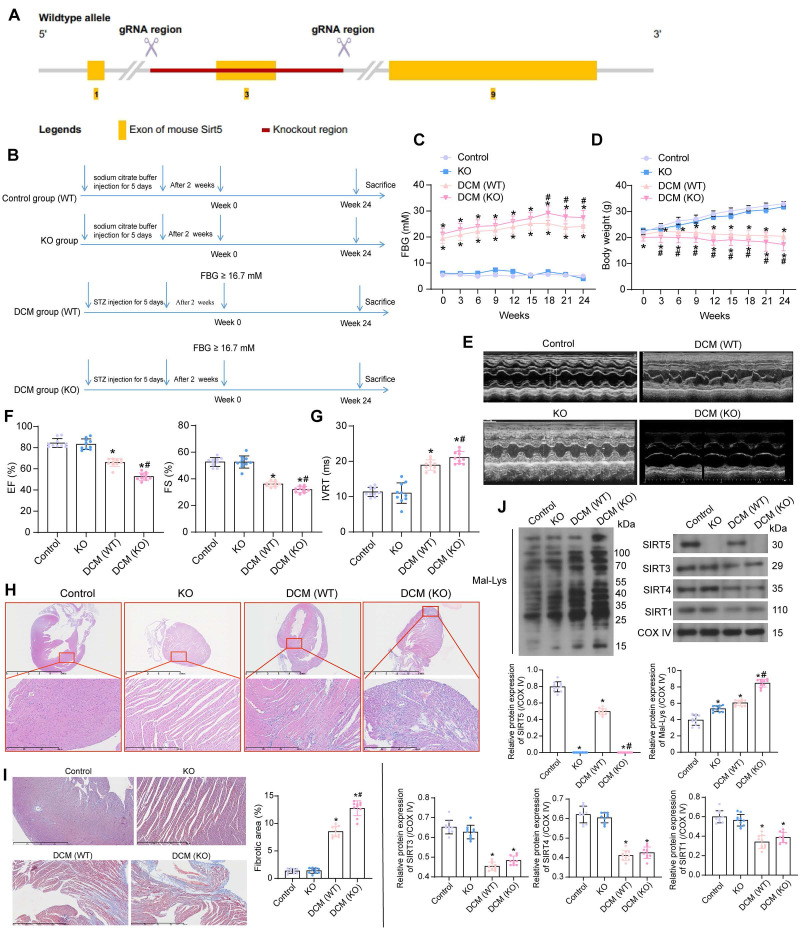
The significance of SIRT5 expression on DCM-related myocardial injury. (A) Strategy for SIRT5 KO mice. (B) Experimental procedure for mice. (C) Changes in FBG levels were assayed every 3 weeks. (D) Changes in body weight were assayed every 3 weeks. (E) M-mode echocardiogram of mice. (F) EF (%) and FS (%) of mice. (G) IVRT of mice. (H) Inflammatory infiltration in mouse myocardial tissues using HE staining. (I) Collagen deposition in myocardial tissues using Masson's staining. (J) The expression of SIRT1, SIRT3, SIRT4, SIRT5, and the Mal-Lys modification of the protein in mouse myocardial tissues using Western blot. All data are expressed as means ± SD (n = 10). *p < 0.05 *vs.* control group; #p < 0.05 *vs.* DCM (WT) group. Continuous data were compared by one-way/two-way ANOVA.

C57BL/6N mice with SIRT5 knockout (KO) were generated by knocking out Exon 3 on SIRT5 (starts from about 12.47% of the coding region and covers 14.41% of the coding region) using CRISPR/Cas9 technology (Fig. 1A). We performed DCM modeling for KO and WT C57BL/6N mice, respectively. WT mice injected with citrate buffer alone served as the normal controls (the control group), and a group of KO mice also received an injection of citrate buffer (the KO group) (Fig. 1B).

There was no significant difference in FBG and body weight between WT and KO mice. DCM-modeled WT mice showed increased FBG levels along with progressive weight loss, and SIRT5 KO mice with DCM exhibited a more pronounced diabetic phenotype (Fig. 1C, D). The cardiac function of mice was assessed at Week 24. Echocardiographic measurements showed no spontaneous cardiac dysfunction in KO mice (Fig. 1E), while a significant decrease in EF (%) and FS (%) (Fig. 1F) and an increase in IVRT were observed (Fig. 1G) in DCM mice. Furthermore, the lack of SIRT5 exacerbated the cardiac impairment in DCM mice. There was no significant difference in heart rate between the control and KO groups. DCM modeling resulted in a significant decrease in heart rate in mice, with SIRT5 KO mice showing a more pronounced decrease in heart rate after DCM modeling (Fig. S1A). The mice in the control and KO groups showed no difference in maximum rate-of-rise of left ventricular pressure (+dP/dt_max_), minimal rate of pressure decay (-dP/dt_min_), early/atrial (E/A), and the ratio of early diastolic transmitral flow velocity to early diastolic mitral annular tissue velocity (E/e'). DCM modeling resulted in decreased +dP/dt_max_ and E/A and elevated -dP/dt_min_ and E/e' in mice, and KO mice exhibited more severe cardiac dysfunction after DCM modeling (Fig. S1B). There was no significant difference in heart weight/tibia length (HW/TL) between control and KO mice, indicating that SIRT5 KO did not cause spontaneous cardiac hypertrophy, whereas DCM modeling significantly increased HW/TL values and SIRT5 KO mice demonstrated more severe cardiac hypertrophy (Fig. S1C). Inflammation and oxidative stress-induced DCM damage are often associated with the overproduction of AGEs 20. The concentration of AGEs in the myocardial tissues of DCM mice was significantly elevated, and the knockout of SIRT5 exacerbated the production of AGEs in the myocardial tissues of DCM mice (Fig. S2A).

HE and Masson's staining of myocardial tissues showed the absence of spontaneous damage to myocardial tissues in KO mice. However, mice in the DCM group exhibited marked disorganization of cardiomyocyte arrangement and inflammatory infiltration with collagen deposition, while SIRT5 KO mice were more sensitive to DCM-related tissue damage (Fig. 1H, I). A decrease in SIRTs (SIRT1, SIRT3, SIRT4, and SIRT5) expression and a significant increase of Mal-Lys modified proteins in myocardial tissues resulting from DCM modeling were detected by Western blot experiments. However, SIRT5 KO mice barely showed SIRT5 expression and a particularly significant increase in Mal-Lys proteins. Meanwhile, no significant change in the expression of other mitochondrial SIRTs (SIRT1, SIRT3, and SIRT4) was caused by SIRT5 KO (Fig. 1J). Through the above experiments, we tentatively demonstrated that reduced expression of SIRT5 in DCM led to DCM-related injury and that Mal-Lys level was elevated in DCM and further enhanced after SIRT5 KO, closely correlating with DCM progression.

### Lack of SIRT5 expression is involved in the progression of HG-induced cardiomyocyte injury

**Figure 2:**
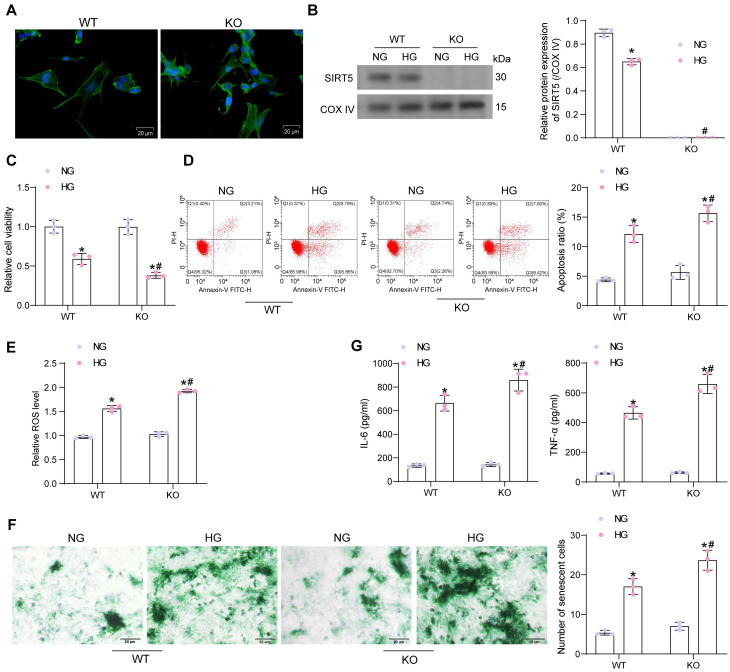
SIRT5 deficiency deteriorates HG-induced cell damage. (A) Immunofluorescence staining of α-Sarcometric actin in primary cardiomyocytes extracted from WT and KO mice. (B) SIRT5 expression in WT and KO cardiomyocytes was examined using Western blot. (C) The viability of WT and KO cardiomyocytes was examined using CCK-8 assay. (D) The apoptosis of WT and KO cardiomyocytes was measured using flow cytometry. (E) Assessment of intracellular ROS levels by DCFH-DA staining. (F) WT and KO cardiomyocyte senescence was detected using β-galactosidase staining. (G) The release of pro-inflammatory factors IL-6 and TNF-α from WT and KO cardiomyocytes using ELISA. All data are expressed as means ± SD (n = 3). *p < 0.05 *vs.* WT:NG or KO:NG group; #p < 0.05 *vs.* WT:HG group. Continuous data were compared by two-way ANOVA.

Primary cardiomyocytes from WT and KO mice were isolated and identified by immunofluorescence of myocyte α-Sarcometric actin (termed as WT and KO, respectively) (Fig. 2A). Cardiomyocytes were treated with HG to simulate DCM injury* in vitro* (with NG as the control). Since SIRT5 is a protein localized in mitochondria, we isolated mitochondria to detect SIRT5 expression using Western blot. HG treatment resulted in decreased SIRT5 expression in the mitochondria of cardiomyocytes from WT mice, while SIRT5 was not expressed in KO cardiomyocytes (Fig. 2B). Cell viability changes were detected by CCK-8 assay. We observed that HG treatment resulted in a decreased viability of both WT and KO cardiomyocytes, and SIRT5-deficient cardiomyocytes were particularly sensitive to HG treatment (Fig. 2C). Flow cytometry was conducted to examine apoptosis in cardiomyocytes exposed to HG. As expected, the lack of SIRT5 further exacerbated the HG-induced apoptosis (Fig. 2D). Also, HG treatment elevated intracellular ROS levels, which were particularly significant in cells with SIRT5 KO (Fig. 2E).

Cellular senescence was detected by β-galactosidase staining. HG upregulated β-galactosidase activity, exhibiting an expanded, flattened, or elongated morphology, while knockdown of SIRT5 deteriorated HG-induced cardiomyocyte senescence (Fig. 2F). By ELISA, we observed HG induced the concentration of pro-inflammatory factors IL-6 and TNF-α in cardiomyocytes, while SIRT5 deficiency further promoted the inflammatory response in cardiomyocytes (Fig. 2G). Therefore, the deficiency of SIRT5 expression resulted in diminished resistance of cardiomyocytes to HG-induced cellular injury, further exacerbating DCM-associated myocardial injury.

### SIRT5 deficiency regulates HG-induced cardiomyocyte pyroptosis and cardiac fibroblast activation

**Figure 3:**
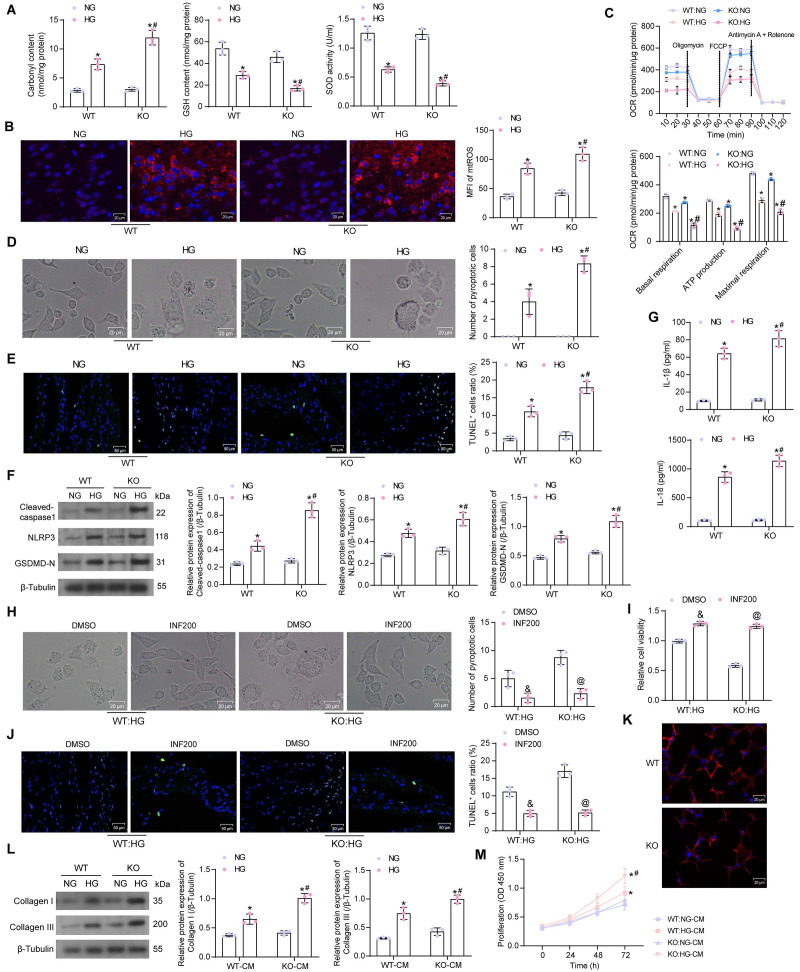
Deficiency of SIRT5 expression exacerbates HG-induced mitochondrial dysfunction and pyroptosis. (A) Effect of HG treatment on the contents of carbonyl, GSH, and SOD in WT and KO cardiomyocytes. (B) Effect of HG treatment on mtROS production in WT and KO cardiomyocytes. (C) The mitochondrial function of WT and KO cardiomyocytes was assessed by seahorse assay. (D) Effect of HG treatment on the pyroptosis of cardiomyocytes from WT and SIRT5 KO mice observed under a light microscope. (E) Effect of HG treatment on DNA damage in cardiomyocytes of WT and SIRT5 KO mice analyzed using TUNEL. (F) Effect of HG treatment on the expression of pyroptosis-related proteins in WT and KO cardiomyocytes using Western blot. (G) Effect of HG treatment on IL-1β and IL-18 release from WT and KO cardiomyocytes using ELISA. (H) Effect of HG and INF200 treatments on the pyroptosis of cardiomyocytes from SIRT5 KO mice observed under a light microscope. (I) Effect of HG and INF200 treatments on viability in cardiomyocytes of SIRT5 KO mice analyzed using TUNEL. (J) Effect of HG and INF200 treatments on DNA damage in cardiomyocytes of SIRT5 KO mice analyzed using TUNEL. (K) The identification of cardiac fibroblasts using Vimentin immunofluorescence staining. (L) Effect of the CM of WT and KO cardiomyocytes exposed to HG on ECM synthesis in cardiac fibroblasts. (M) Effect of the CM of WT and KO cardiomyocytes exposed to HG on the proliferative capacity of cardiac fibroblasts. All data are expressed as means ± SD (n = 3). *p < 0.05 *vs.* WT:NG or KO:NG group; #p < 0.05 *vs.* WT:HG group; &p < 0.05 *vs.* WT:HG:DMSO group; @p < 0.05 *vs.* KO:HG:DMSO group. Continuous data were compared by two-way ANOVA.

It was found by ELISA that HG elevated Carbonyl content in cardiomyocytes, while diminished GSH and SOD contents. The cellular levels of oxidative stress were further amplified in SIRT5-deficient cardiomyocytes (Fig. 3A). The mtROS production was observed using MitoSox Red staining. HG treatment also induced mitochondrial damage in cardiomyocytes, leading to increased mtROS production, which was exacerbated by SIRT5 deletion (Fig. 3B). By OCR analysis, it was observed that SIRT5 knockout resulted in mitochondrial dysfunction. Basal respiration, ATP production, and maximal respiration were all diminished in cardiomyocytes, and mitochondrial dysfunction was more pronounced under the HG context following SIRT5 KO (Fig. 3C).

Light microscopy observation showed that HG induced swollen and expanded cells and numerous bubble-like protrusions, while SIRT5 knockout significantly increased the number of pyroptotic cardiomyocytes (Fig. 3D). An increase in the number of cardiomyocytes with DNA damage (TUNEL^+^) caused by HG treatment was observed by TUNEL, whereas the knockout of SIRT5 exacerbated DNA damage in HG-induced cardiomyocytes (Fig. 3E). The expression of Cleaved-caspase1, NLRP3, and GSDMD-N was significantly increased in HG-treated cardiomyocytes, and SIRT5 deletion further upregulated the expression of pyroptosis-related proteins in the cells (Fig. 3F). Consistently, we also detected a significant increase in IL-1β and IL-18 levels in the HG-induced cardiomyocyte supernatant, while SIRT5 deficiency promoted the secretion of pro-inflammatory factors (Fig. 3G). To analyze whether HG-induced cardiomyocyte injury enhanced by knockout of SIRT5 was dependent on the pyroptosis pathway, we treated cardiomyocytes in the KO group with the pyroptosis inhibitor INF200. It was observed under the light microscope that INF200 successfully blocked the HG cardiomyocyte pyroptosis exacerbated by SIRT5 knockout, and there was no significant difference in the pyroptosis of cells in both WT and KO groups after HG and INF200 treatments (Fig. 3H). CCK-8 and TUNEL analyses showed that INF200 significantly ameliorated the diminished viability and DNA damage in cardiomyocytes by blocking cell pyroptosis exacerbated by SIRT5 knockout, and there was no significant difference between the damage levels of WT and KO cells in the two groups (Fig. 3I, J). This suggests that HG cardiomyocyte injury exacerbated by knockout of SIRT5 is closely related to the pyroptosis pathway and that the cardioprotective effect of SIRT5 is not independent of pyroptosis.

We prepared WT mouse-derived cardiac fibroblasts, followed by the identification of Vimentin immunofluorescence (Fig. 3K). The conditioned medium (CM) of HG-treated cardiomyocytes significantly stimulated cardiac fibroblast activity, leading to the deposition of ECM-associated proteins Collagen I and III (Fig. 3L) and proliferation of fibroblasts (Fig. 3M), while SIRT5-deficient cells induced stronger cardiac fibroblast activity (Fig. 3L, M).

### GSTP1 is a downstream target of SIRT5 in DCM

**Figure 4:**
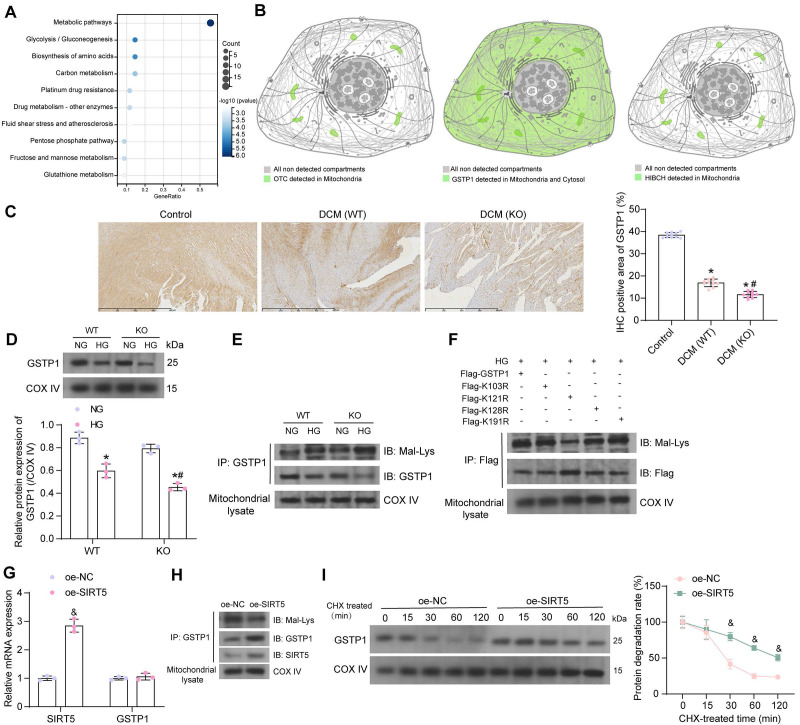
SIRT5 stabilizes the GSTP1 protein via lysine demalonylation. (A) KEGG pathway enrichment analysis of proteins with significantly elevated Mal-Lys modifications in SIRT5 KO mice. (B) OTC, GSTP1, and HIBCH are expressed in mitochondria. (C) Immunohistochemical detection of GSTP1 expression in the myocardial tissues of mice. (D) GSTP1 expression in mitochondria of HG-treated WT and KO cardiomyocytes using Western blot. (E) Mal-Lys modification of GSTP1 protein in mitochondria of HG-treated WT and KO cardiomyocytes using Western blot. (F) Validation of Mal-Lys modification site of GSTP1 using Western blot. (G) Effect of oe-SIRT5 on SIRT5 and GSTP1 mRNA expression by RT-qPCR. (H) Effect of overexpression of SIRT5 on Mal-Lys modification of GSTP1 and its protein expression using Western blot. (I) Effect of overexpression of SIRT5 on the stability of GSTP1 protein. All data are expressed as means ± SD (n = 3 or 10). *p < 0.05 *vs.* Control, WT:NG or KO:NG group; #p < 0.05 *vs.* WT:HG group, &p < 0.05 *vs.* oe-NC group. Continuous data were compared by one-way or two-way ANOVA.

To reveal the molecular mechanisms underlying the ameliorating effects of SIRT5 on cardiomyocyte injury, we conducted a literature review. Proteins with significantly elevated Mal-Lys modification in SIRT5 KO mice (*p-value* < 0.05) from Nishida *et al.* 13 were subjected to KEGG enrichment analysis. We found that the vast majority of proteins were enriched to hsa01100: Metabolic pathways (Fig. 4A). Given that SIRT5 is a mitochondrial-localized protein, we analyzed proteins enriched to Metabolic pathways, including ALDOB, GNMT, PRDX6, OTC, ALDOA, GSTP1, PGAM1, DPYS, GSTA3, ADK, RPN1, ACSL1, KHK, GSTM1, PGK1, HIBCH, TECR, UGDH, PGM1 for subcellular localization using THE HUMAN PROTEIN ATLAS (https://www.proteinatlas.org/). Only OTC, GSTP1, and HIBCH showed protein expression in mitochondria (Fig. 4B). The GSTP family protects cells from exogenous and endogenous oxidative stress 21. Polymorphisms in the GSTP1 gene are associated with susceptibility to diabetic nephropathy 22. However, the effect of post-translational modification of this protein on DCM has not been reported.

By immunohistochemistry, we detected a significant decrease in GSTP1 expression in the myocardial tissues of DCM mice, and the deletion of SIRT5 further downregulated GSTP1 protein expression (Fig. 4C). HG reduced GSTP1 protein expression in the mitochondria of cardiomyocytes, and SIRT5 deletion exacerbated the GSTP1 protein loss. Interestingly, GSTP1 showed a degree of reduction in the NG-treated KO cardiomyocytes, but the difference was insignificant compared to the NG-treated WT cardiomyocytes, suggesting that SIRT5 significantly affected GSTP1 protein expression only under HG conditions (Fig. 4D). Further studies revealed significantly increased Mal-Lys modification of GSTP1 protein in mitochondria of HG-induced cardiomyocytes, and this modification was particularly pronounced in the absence of SIRT5 (Fig. 4E). This suggests that SIRT5 controls lysine demalonylation function only when HG induces the Mal-Lys modification of GSTP1 protein.

To minimize animal sacrifice, we used WT mice and their source cells for functional validation. We predicted the Mal-Lys modification site on GSTP1 (UniProt accession: P19157) using Mal-Lys 23, and there are four potential Mal-Lys modification sites for the 12 lysines (Table 2). The fusion proteins K103R, K121R, K128R, K191R, and wild-type Flag-GSTP1 were designed for recombinant arginine (R) mutations of lysine (K) at different loci with Flag tags.

To explore which potential Mal-Lys modification site is the site involved in Mal-Lys modification in cardiomyocytes, the plasmids were transfected into WT mouse cardiomyocytes and treated with HG. By western blot analysis, we observed that the K121 mutation diminished Mal-Lys modification and elevated protein expression on GSTP1, indicating that this site is the major functional modification site (Fig. 4F). The oe-SIRT5 and oe-NC plasmids were transfected into WT mouse cardiomyocytes, respectively, and treated with HG. Using RT-qPCR, we detected that oe-SIRT5 significantly increased the expression of SIRT5 mRNA in HG-treated cardiomyocytes without affecting GSTP1 mRNA expression (Fig. 4G). Moreover, overexpression of SIRT5 increased the interaction of SIRT5 with GSTP1 protein, as evidenced by elevated expression of SIRT5 and GSTP1 in the GSTP1 pull-down protein, while the level of Mal-Lys modification of GSTP1 protein was decreased (Fig. 4H). The results of the protein stability assay showed that overexpression of SIRT5 significantly reduced the degradation rate of GSTP1 protein and enhanced its stability (Fig. 4I).

### SIRT5 alleviates HG-induced cardiomyocyte injury by promoting GSTP1 protein expression

**Figure 5:**
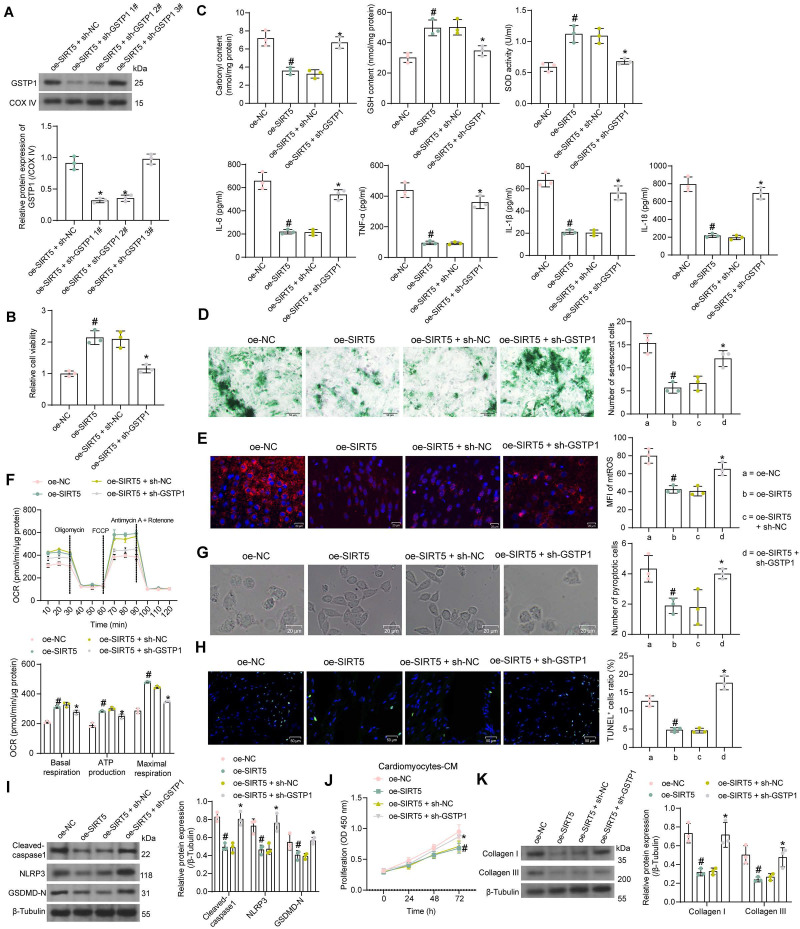
SIRT5-controlled GSTP1 mediates HG-induced cardiomyocyte injury. (A) The effect of sh-GSTP1 on GSTP1 protein expression using Western blot. WT cardiomyocytes were transfected with oe-SIRT5 alone (with oe-NC as control) or in combination with sh-GSTP1 (with oe-SIRT5 + sh-NC as control), followed by HG. (B) The viability of HG-treated cardiomyocytes was examined using a CCK-8 assay. (C) The oxidative stress levels and inflammatory factor release in HG-treated cardiomyocytes using ELISA. (D) HG-treated cardiomyocyte senescence was detected using β-galactosidase staining. (E) The mtROS production in HG-treated cardiomyocytes using MitoSox Red staining. (F) The mitochondrial function of HG-treated cardiomyocytes was assessed by seahorse assay. (G) The pyroptosis of cardiomyocytes from WT and SIRT5 KO mice was observed under a light microscope. (H) DNA damage in cardiomyocytes treated with HG was analyzed using TUNEL. (I) The expression of pyroptosis-related proteins in HG-treated cardiomyocytes using Western blot. (J) Effect of the CM of cardiomyocytes after transfection and exposure to HG on the proliferative capacity of cardiac fibroblasts. (K) Effect of cardiomyocytes after transfection and exposure to HG on ECM synthesis in cardiac fibroblasts. All data are expressed as means ± SD (n = 3). *p < 0.05 *vs.* oe-SIRT5 + sh-NC group; #p < 0.05 *vs.* oe-NC group. Continuous data were compared by one-way or two-way ANOVA.

Since there are no means to specifically reduce the stability of GSTP1 protein, we transfected three shRNAs targeting GSTP1 into cardiomyocytes overexpressing SIRT5 to verify whether the function of SIRT5 is dependent on promoting GSTP1 protein expression. We detected that sh-GSTP1 1# and 2# significantly decreased the protein expression of GSTP1 using the Western blot experiment, while the repressive effect of sh-GSTP1 3# was not significant (Fig. 5A). The sh-GSTP1 1# with the best inhibitory effect was selected as the shRNA of GSTP1 for the following experiments (termed as sh-GSTP1).

By CCK-8 assay we detected that overexpression of SIRT5 increased the activity of HG-treated cardiomyocytes, while cell viability was significantly reduced after suppression of GSTP1 (Fig. 5B). HG-induced oxidative stress levels and inflammatory factor release in cardiomyocytes were significantly inhibited by SIRT5 overexpression, while inhibition of GSTP1 attenuated the protective effect of SIRT5 (Fig. 5C). SIRT5 overexpression also ameliorated HG-induced cellular senescence (Fig. 5D), inhibited mtROS generation (Fig. 5E), and enhanced mitochondrial respiratory function (Fig. 5F), which were dependent on GSTP1 expression. Light microscopy observed that SIRT5 reduced pyroptosis, and the knockdown of GSTP1 resulted in the loss of pyroptosis inhibition by SIRT5 (Fig. 5G). Consistently, it was found using the TUNEL assay that SIRT5 ameliorated HG-induced DNA damage, whereas inhibition of GSTP1 blocked the protective effect of SIRT5 against DNA damage (Fig. 5H). The expression of Cleaved-caspase1, NLRP3, and GSDMD-N were downregulated by SIRT5, while simultaneous knockdown of GSTP1 resulted in a restoration of expression of pyroptosis-associated proteins (Fig. 5I).

Further experiments showed that the pro-proliferative effect of cardiomyocyte-CM on cardiac fibroblasts was significantly attenuated after overexpression of SIRT5, which was again reversed by inhibition of GSTP1 (Fig. 5J). The accumulation of ECM-related proteins induced by HG was alleviated by overexpression of SIRT5 and promoted again by GSTP1 knockdown (Fig. 5K).

The GSTP1-K121R mutant plasmid was combined with shRNAs targeting SIRT5 to further validate that the protective effect of SIRT5 on cardiomyocytes was dependent on GSTP1. Transfection with oe-GSTP1-K121R significantly increased GSTP1 expression in HG-treated cardiomyocytes, and SIRT5 shRNA-mediated SIRT5 knockdown was unable to significantly affect GSTP1 protein expression (Fig. S3A). sh-SIRT5 1# was chosen for subsequent experiments. Overexpression of mutant GSTP1 also alleviated HG-repressed cardiomyocyte viability (Fig. S3B), significantly inhibited senescence in HG-treated cardiomyocytes (Fig. S3C), suppressed mtROS production (Fig. S3D), improved mitochondrial respiratory function (Fig. S3E), and suppressed expression of pyroptosis-related proteins (Fig. S3F). In contrast, the knockdown of SIRT5 failed to significantly alter the protective effect of GSTP1-K121R on cardiomyocytes (Fig. S3B-S3F). Transfection of oe-GSTP1-K121R inhibited cardiac fibroblast proliferation and reduced ECM-associated protein accumulation, and simultaneous knockdown of SIRT5 in cardiomyocytes failed to reverse the function of GSTP1-K121R (Fig. S3G-S3H).

The above experiments show that K121 is a SIRT5-mediated Mal-Lys modification site, but does not alter the function of GSTP1. Knockdown of SIRT5 may inhibit endogenous GSTP1 expression, but compared to the lower endogenous GSTP1 expression under HG treatment (Fig. 4D), exogenous GSTP1-K121R-mediated protein expression was significant (Fig. S3A). GSTP1-K121R, which could not be inhibited by SIRT5 knockdown, significantly rescued the endogenous protein inhibited by knockdown of SIRT5, resulting in the inability of SIRT5 knockdown to reverse the protective effect of GSTP1-K121R on cardiomyocytes.

### SIRT5 ameliorates myocardial injury in DCM mice by promoting the expression of GSTP1

**Figure 6:**
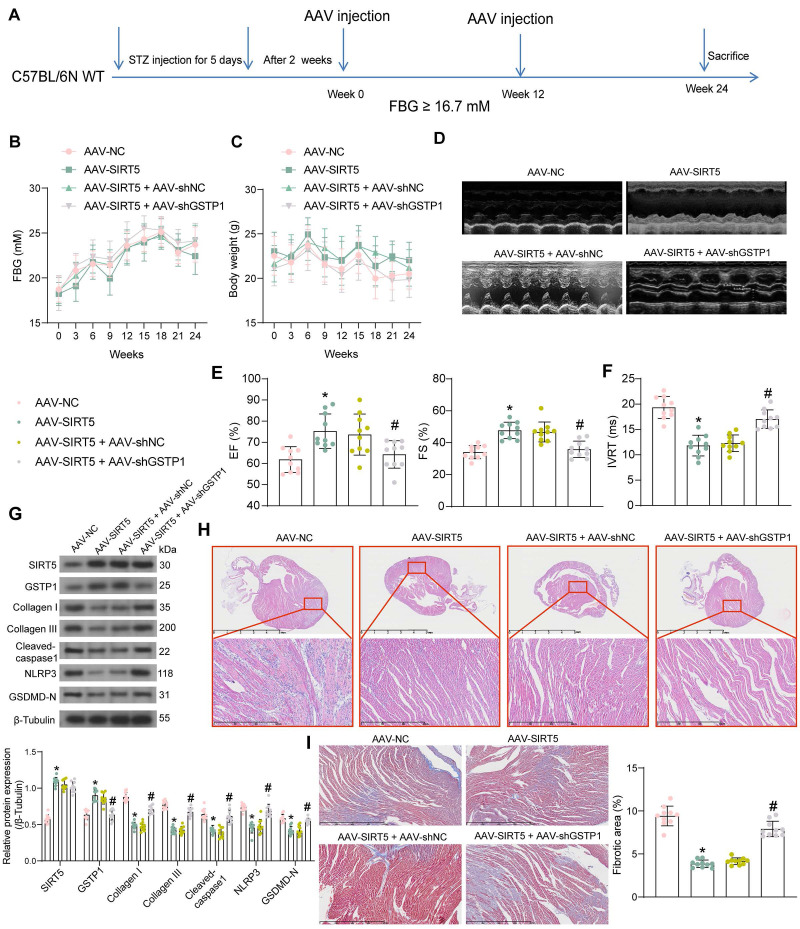
SIRT5-mediated GSTP1 overexpression alleviates myocardial injury in DCM mice. (A) Schematic diagram of the DCM mouse model and the AAV treatment schedule. (B) Changes in FBG levels were assayed every 3 weeks. (C) Changes in body weight were assayed every 3 weeks. (D) M-mode echocardiogram of mice. (E) EF (%) and FS (%) of mice. (F) IVRT of mice. (G) The protein expression of SIRT5, GSTP1, Cleaved-caspase1, NLRP3, GSDMD-N, and Collagen I and III in mouse myocardial tissues using Western blot. (H) Inflammatory infiltration in mouse myocardial tissues using HE staining. (I) Collagen deposition in myocardial tissues using Masson's staining. All data are expressed as means ± SD (n = 10). *p < 0.05 *vs.* AAV-NC group; #p < 0.05 *vs.* AAV-SIRT5 + AAV-shNC group. Continuous data were compared by one-way/two-way ANOVA.

We performed DCM modeling in WT C57BL/6N mice and *in situ* injection (Fig. 6A) of AAV-SIRT5 alone or in combination with AAV-shGSTP1 (shRNA sequence sh-GSTP1 1#). We observed no significant effect of AAV treatment on blood glucose and body weight of DCM mice, suggesting that the cardiomyocyte tropism of AAV was outstanding (Fig. 6B, C). In contrast, echocardiographic measurements showed significantly higher EF (%) and FS (%) and shorter IVRT in AAV-SIRT5-treated DCM mice, and AAV-shGSTP1 administration resulted in a reduced therapeutic effect of AAV-SIRT5 (Fig. 6D-F).

AAV-SIRT5-treated DCM mice showed a significant increase in heart rate (Fig. S1D) and an increase in +dP/dt_max_ and E/A and a decrease in -dP/dt_min_ and E/e' (Fig. S1E), indicating that AAV-SIRT5 alleviated diastolic function, whereas the combination of AAV-shGSTP1 resulted in a diminished therapeutic effect of AAV-SIRT5 (Fig. S1D-S1E). AAV-SIRT5 also improved cardiac hypertrophy in mice with a significant reduction in HW/TL, whereas AAV-shGSTP1 treatment restored the HW/TL value (Fig. S1F). AAV-SIRT5 decreased the concentration of AGEs in myocardial tissues, whereas AAV-shGSTP1 increased the production of AGEs (Fig. S2B).

The expression of SIRT5, GSTP1, Cleaved-caspase1, NLRP3, GSDMD-N, and Collagen I and III in tissues was detected by Western blot (Fig. 6G). AAV-SIRT5 appreciably augmented the protein expression of SIRT5 and GSTP1, and inhibited the expression of Cleaved-caspase1, NLRP3, GSDMD-N, and Collagen I and III. Combined application of AAV-shGSTP1 did not significantly affect the expression of SIRT5 but significantly inhibited the expression of GSTP1 and elevated pyroptosis- and ECM-related proteins. Overexpression of SIRT5 was observed to significantly inhibit inflammatory cell infiltration and suppress collagen deposition in the myocardial tissues of DCM mice by HE and Masson's trichrome staining, while the protective effect of SIRT5 on tissues was significantly attenuated after inhibition of GSTP1 expression (Fig. 6H, I).

### SPI1 loss in DCM is responsible for the SIRT5 deletion

**Figure 7:**
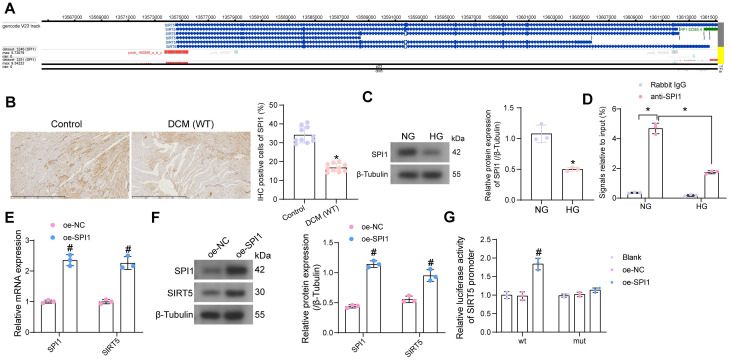
SPI1 promotes the transcriptional expression of SIRT5. (A) SPI1 targets the SIRT5 promoter region in cardiac tissue samples. (B) Detection of SPI1 expression in the myocardial tissues of DCM mice by immunohistochemical assay. (C) The effect of HG treatment on SPI1 protein expression in WT cardiomyocytes using Western blot. (D) The enrichment ability of SPI1 on SIRT5 promoter using ChIP-qPCR. (E) The effect of overexpression of SPI1 on SPI1 and SIRT5 mRNA expression in WT cardiomyocytes using RT-qPCR. (F) The effect of overexpression of SPI1 on SPI1 and SIRT5 protein expression in WT cardiomyocytes using Western blot. (G) The effect of overexpression of SPI1 on the transcriptional activity of SIRT5 promoter using a dual-luciferase reporter assay. All data are expressed as means ± SD (n = 10 or 3). *p < 0.05 *vs.* Control, NG, IgG group; #p < 0.05 *vs.* oe-NC group. Continuous data were compared by unpaired t-test or two-way ANOVA.

We have demonstrated that SIRT5 expression is reduced in the myocardium of DCM mice and that SIRT5 ameliorates myocardial injury in DCM through its downstream functional target GSTP1. However, the cause of the reduced SIRT5 expression in myocardial tissues is still unclear. Using hTFtarget (http://bioinfo.life.hust.edu.cn/hTFtarget/#!/), we predicted transcription factors that could target SIRT5, of which SPI1 targeted SIRT5 in myocardial tissues (Fig. 7A).

To investigate whether the low expression of SIRT5 in DCM was associated with the expression of SPI1, we first detected its expression in myocardial tissues of DCM mice and HG-exposed cardiomyocytes using immunohistochemistry and Western blot, respectively (Fig. 7B, C). The expression of SPI1 was considerably downregulated in the DCM or HG conditions. By ChIP-qPCR experiments, we detected that SPI1 significantly enriched the promoter sequence of SIRT5, and the enrichment of the SIRT5 promoter sequence by SPI1 was significantly reduced under HG treatment (Fig. 7D). To verify whether SPI1 regulates SIRT5 expression in cardiomyocytes, we transfected SPI1 overexpression DNA plasmid into cardiomyocytes, followed by HG treatment. RT-qPCR and Western blot results showed that overexpression of SPI1 significantly increased SIRT5 mRNA and protein expression in cardiomyocytes (Fig. 7E, F). Furthermore, overexpression of SIRT5 significantly increased the transcriptional activity of the wild type SIRT5 promoter sequence and had no significant effect on the transcriptional activity of the mutant SIRT5 promoter, as judged by a dual-luciferase assay (Fig. 7G).

### SPI1 exerts an inhibitory effect on cardiomyocyte oxidative stress and pyroptosis by promoting the transcription of SIRT5

**Figure 8:**
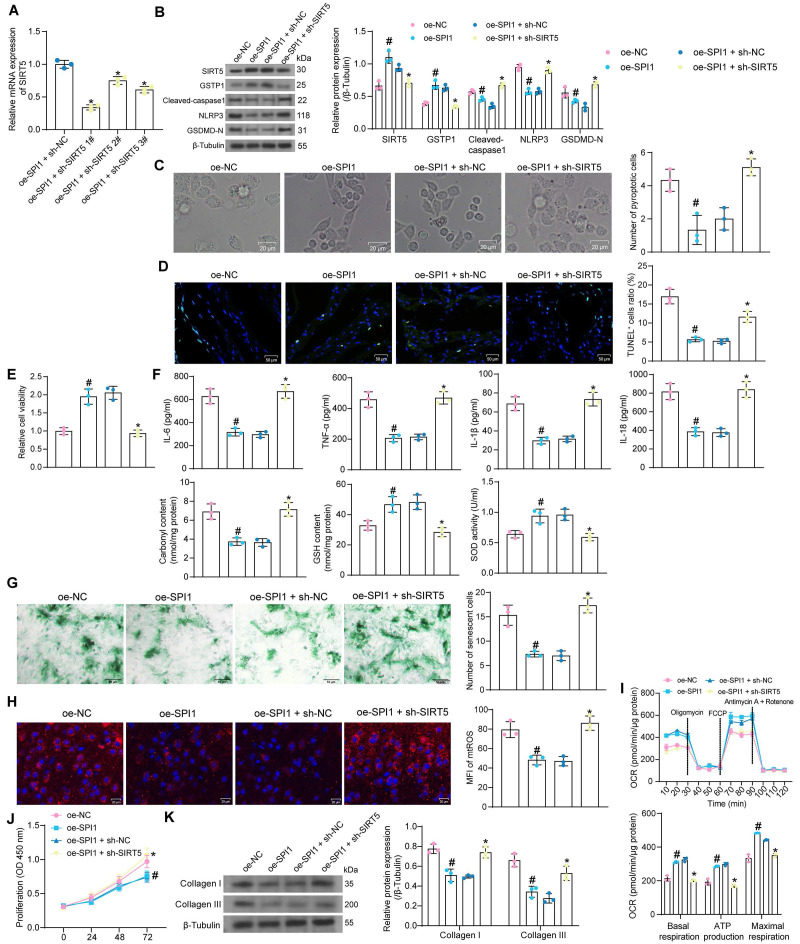
SPI1-mediated transcriptional activation of SIRT5 inhibits HG-induced cardiomyocyte injury. (A) Interference efficiency of sh-SIRT5 in cardiomyocytes by RT-qPCR. WT cardiomyocytes were transfected with oe-SPI1 alone (with oe-NC as control) or in combination with sh-SIRT5 (with oe-SPI1 + sh-NC as control), followed by HG. (B) The expression of SIRT5, GSTP1, and pyroptosis-related proteins in HG-treated cardiomyocytes using Western blot. (C) The pyroptosis of HG-treated cardiomyocytes was observed under a light microscope. (D) DNA damage in HG-treated cardiomyocytes was analyzed using TUNEL. (E) The viability of HG-treated cardiomyocytes was examined using CCK-8 assays. (F) The oxidative stress levels and inflammatory factor release in HG-treated cardiomyocytes using ELISA. (G) HG-treated cardiomyocyte senescence was detected using β-galactosidase staining. (H) The mtROS production in HG-treated cardiomyocytes using MitoSox Red staining. (I) The mitochondrial function of HG-treated cardiomyocytes was assessed by seahorse assay. (J) Effect of the CM of cardiomyocytes after transfection and exposure to HG on the proliferative capacity of cardiac fibroblasts. (K) Effect of cardiomyocytes after transfection and exposure to HG on ECM synthesis in cardiac fibroblasts. All data are expressed as means ± SD (n = 3). *p < 0.05 *vs.* oe-SPI1 + sh-NC group; #p < 0.05 *vs.* oe-NC group. Continuous data were compared by one-way or two-way ANOVA.

We co-transfected the shRNA sequences of SIRT5 into cardiomyocytes overexpressing SPI1. HG treatment was then applied to transfected cells. RT-qPCR results showed that sh-SIRT5 1# produced the most outstanding interference effect and was selected as the shRNA sequence of SIRT5 for subsequent experiments (Fig. 8A).

We detected that overexpression of SPI1 increased the protein expression of SIRT5 and GSTP1 and suppressed the expression of pyroptosis-related proteins in HG-treated cardiomyocytes using Western blot assays. However, inhibition of SIRT5 resulted in a decrease in GSTP1 expression and restoration of pyroptosis-related proteins (Fig. 8B). Overexpression of SPI1 significantly reduced HG-induced pyroptosis and ameliorated DNA damage, whereas inhibition of SIRT5 reversed the protective effect of SPI1 (Fig. 8C, D). Cardiomyocyte viability was significantly increased after overexpression of SPI1, while the promoting effect of SPI1 on cell viability was significantly reversed after inhibition of SIRT5 (Fig. 8E). HG-induced inflammatory responses and cellular oxidative stress were significantly attenuated by overexpression of SPI1, while the associated damage in cells was enhanced after inhibition of SIRT5 (Fig. 8F). Overexpression of SPI1 inhibited HG-induced cardiomyocyte senescence and mtROS generation, whereas inhibition of SIRT5 resulted in an increased number of senescent cells and mtROS generation (Fig. 8G, H). OCR analysis showed that overexpression of SPI1 significantly improved mitochondrial respiratory function in cardiomyocytes, while inhibition of SIRT5 significantly reduced basal respiration, maximal respiration, and ATP production (Fig. 8I). SPI1 overexpression also inhibited the proliferation of cardiac fibroblasts induced by cardiomyocyte-CM in the HG condition and reduced the expression of ECM-related proteins in cardiac fibroblasts. After inhibition of SIRT5 in cardiomyocytes, fibroblasts were reactivated (Fig. 8J, K).

### SPI1-mediated activation of SIRT5/GSTP1 axis ameliorates DCM-associated myocardial injury

**Figure 9:**
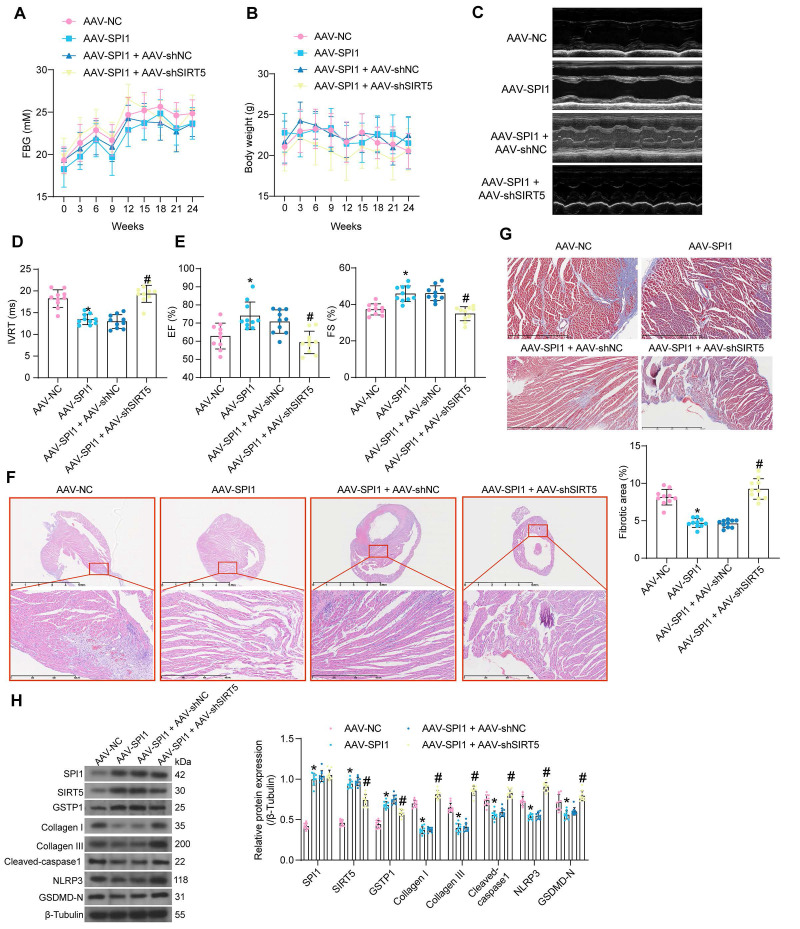
Protective effect of SPI1 on myocardial injury in DCM mice is elicited through the activation of SIRT5. DCM mice were further administrated with AAV-SPI1 (AAV-NC as control) or in combination with AAV-shSIRT5 (AAV-SPI1 + AAV-shNC as control). (A) Changes in FBG levels were assayed every 3 weeks. (B) Changes in body weight were assayed every 3 weeks. (C) M-mode echocardiogram of mice. (D) IVRT of mice. (E) EF (%) and FS (%) of mice. (F) Inflammatory infiltration in mouse myocardial tissues using HE staining. (G) Collagen deposition in myocardial tissues using Masson's staining. (H) The protein expression of SPI1, SIRT5, GSTP1, Cleaved-caspase1, NLRP3, GSDMD-N, and Collagen I and III in mouse myocardial tissues using Western blot. All data are expressed as means ± SD (n = 10). *p < 0.05 *vs.* AAV-NC group; #p < 0.05 *vs.* AAV-SPI1 + AAV-shNC group. Continuous data were compared by one-way/two-way ANOVA.

AAV was used to intervene in the expression of SPI1 and SIRT5 in cardiomyocytes of DCM mice according to the previously described procedure. AAV treatment did not significantly affect FBG and body weight (Fig. 9A, B). AAV-SPI1 significantly improved cardiac function in DCM mice, as evidenced by reduced IVRT and increased EF (%) and FS (%), while AAV-shSIRT5 reversed the promoting effect of AAV-SPI1 on cardiac function (Fig. 9C-E).

AAV-SPI1 treatment rescued the decrease in heart rate in DCM mice (Fig. S1G), and increased +dP/dt_max_ and E/A and decreased -dP/dt_min_ and E/e' (Fig. S1H), while AAV-shSIRT5 treatment resulted in loss of the cardioprotective effect of AAV-SPI1 (Fig. S1G-S1H). AAV-SPI1 treatment resulted in a significant decrease in HW/TL, whereas AAV-shSIRT5 treatment restored HW/TL (Fig. S1I). AAV-SPI1 inhibited the production of AGEs in myocardial tissues, whereas AAV-shSIRT5 led to a restoration in AGEs concentration (Fig. S2C).

We observed inhibitory effects of AAV-SPI1 on inflammatory damage (Fig. 9F) and collagen deposition (Fig. 9G) in myocardial tissues using HE and Masson's staining, and these inhibitory effects could be significantly reversed by AAV-shSIRT5 administration. Finally, Western blot experiments showed that AAV-SPI1 increased protein expression of SIRT5 and GSTP1 and suppressed expression of pyroptosis and collagen production-related proteins, while AAV-shSIRT5 suppressed GSTP1 expression by decreasing SIRT5 expression and reactivated pyroptosis and collagen production in tissues (Fig. 9H).

## Discussion

Since a strategy for prevention and treatment to improve the prognosis of DCM has not been well-established, it is vital to identify pathophysiological landmarks and to spotlight potential therapeutic targets 24. There were several important findings in the present study. First, SIRT5 KO mice with DCM showed cardiac dysfunction and inflammatory infiltration and collagen deposition in the myocardial tissues, while primary cardiomyocytes extracted from SIRT5 KO mice exhibited mitochondrial dysfunction and pyroptosis, leading to activation of cardiac fibroblasts. Second, GSTP1 was a possible downstream target of SIRT5-mediated lysine demalonylation. Third, the cardioprotective effects of SIRT5 were abolished by a combined administration of shRNA targeting GSTP1. Fourth, SIRT5 downregulation in DCM was attributed to SPI1 loss. Finally, SIRT5 inhibition reversed the beneficial effects of oe-SPI1 administration *in vitro* and *in vivo*.

Subarachnoid hemorrhage has been reported to decrease SIRT5 expression and succinylated citrate synthase as well as the subunits of ATP synthase, subsequently increasing ROS production, leading to neuronal cell death and neurological deficits 25. Here, it was also noted that DCM modeling contributed to lowered SIRT5 in the myocardial tissues, along with enhanced proteins modified by Mal-Lys. SIRT5 is principally located in the mitochondria and is overexpressed in the brain, heart, testis, and lymphoblasts 26. Mitochondrial SIRTs coordinate the modulation of gene expression and activities of a myriad of enzymes to orchestrate oxidative metabolism and stress responses 27. Consistently, we found the downregulation of four SIRTs in the myocardial tissues of mice with DCM. Even though seven sirtuin family proteins (SIRT1-7) have been identified as mammalian SIR2 orthologs and related to cardiac disease, the roles of SIRT4 and SIRT5 in the heart remain largely uncharacterized 28. Intriguingly, SIRT5 mediated desuccinylation of p53 at K120, resulting in the suppression of p53 activation 29. It has been recently reported that Neuraminidase 1 inhibition attenuated HG-induced ROS generation, inflammation, and cardiomyocyte death *in vitro*, which was blocked by SIRT3 deficiency 30. In the present study, we observed that besides the direct effects on cardiomyocyte viability and senescence, SIRT5 depletion also contributed to mitochondrial damage and pyroptosis in cardiomyocytes.

Pyroptosis is characterized by GSDMD- or GSDME-mediated necrosis with excessive inflammatory factor release, and the N-terminal fragment of GSDMD is required for cardiomyocyte pyroptosis 31. Long noncoding RNA KLF3-AS1 in human mesenchymal stem cell-derived exosomes ameliorated pyroptosis of cardiomyocytes and myocardial infarction through upregulating SIRT1 32. However, the regulatory role of SIRT5 in pyroptosis remains unclear. We observed that SIRT5 deletion further amplified the expression of Cleaved-caspase1, GSDMD-N, and NLRP3 induced by HG in cardiomyocytes. Cardiac fibroblasts, the primary matrix-producing cells in the myocardium, can be activated by mechanical or bioactive pathological insults, characterized by heightened proliferation, migration, contractility, and ECM production 33. Our co-culture system using CM from HG-treated cardiomyocytes stimulated the activation of cardiac fibroblasts, as evidenced by stronger proliferation and ECM production, and the active state was more pronounced when the cardiomyocytes were extracted from SIRT5 KO mice.

As we mentioned above, lysine malonylation is a significant post-translational modification in proteins and has been characterized to be associated with various diseases 23. Quercetin, an ingredient in many medicinal plants, has been revealed to stimulate the desuccinylation of IDH2 via SIRT5, thus maintaining mitochondrial homeostasis, protecting mouse cardiomyocytes against inflammatory responses, alleviating myocardial fibrosis, and eventually diminishing the incidence of heart failure 34. SIRT5 is a NAD-dependent protein lysine demalonylase and desuccinylase 35. However, whether it can affect HG-induced cardiomyocyte injury by demalonylation modification and whether SIRT5 affects myocardial injury in DCM mice have not been studied. Our study here not only identified GSTP1 as a downstream target of SIRT5-mediated lysine demalonylation but also presented that the cardioprotective effects of SIRT5 were elicited through GSTP1. Increased hypermethylation of GSTP1, a potential biomarker, has been implicated in the progression of prostate cancer 36. Moreover, Balchin *et al.* found that S-nitrosation, another post-translational modification, at Cys47 and Cys101 has been revealed to reduce the activity of GSTP1 by 94% 37. These findings suggested that the expression of GSTP1 was widely regulated by post-transcriptional and post-translational modifications under pathological conditions. As for its functional role, Conklin *et al* have shown that GSTP1 protects the heart from ischemia-reperfusion injury by facilitating the detoxification of cytotoxic aldehydes 38. Since three proteins were found to be targets of SIRT5 and we only probed the role of GSTP1, further studies regarding other targets are necessary to deepen the function of SIRT5.

We subsequently switched to the upstream mechanism for SIRT5 in DCM. It was found that the loss of transcription factor SPI1 was responsible for the deletion of SIRT5 in the current work. MicroRNA-223-3p promoted pyroptosis of cardiomyocytes and the release of inflammasome factors by reducing the SPI1 expression 39. Our rescue experiments also demonstrated that overexpression of SPI1 showed similar therapeutic effects *in vitro* and* in vivo* as overexpression of SIRT5, and these effects were compromised by silencing of SIRT5. This study has some limitations. First, the impact of SIRT5 on protein lysine malonylation in myocardial tissues was examined using Western blot, and mass spectrometry was not performed to detect all proteins with altered levels of lysine malonylation. Second, the WT mice and KO mice used in this study came from different sources, and littermate mice will be used as controls for our following study.

**Figure 10:**
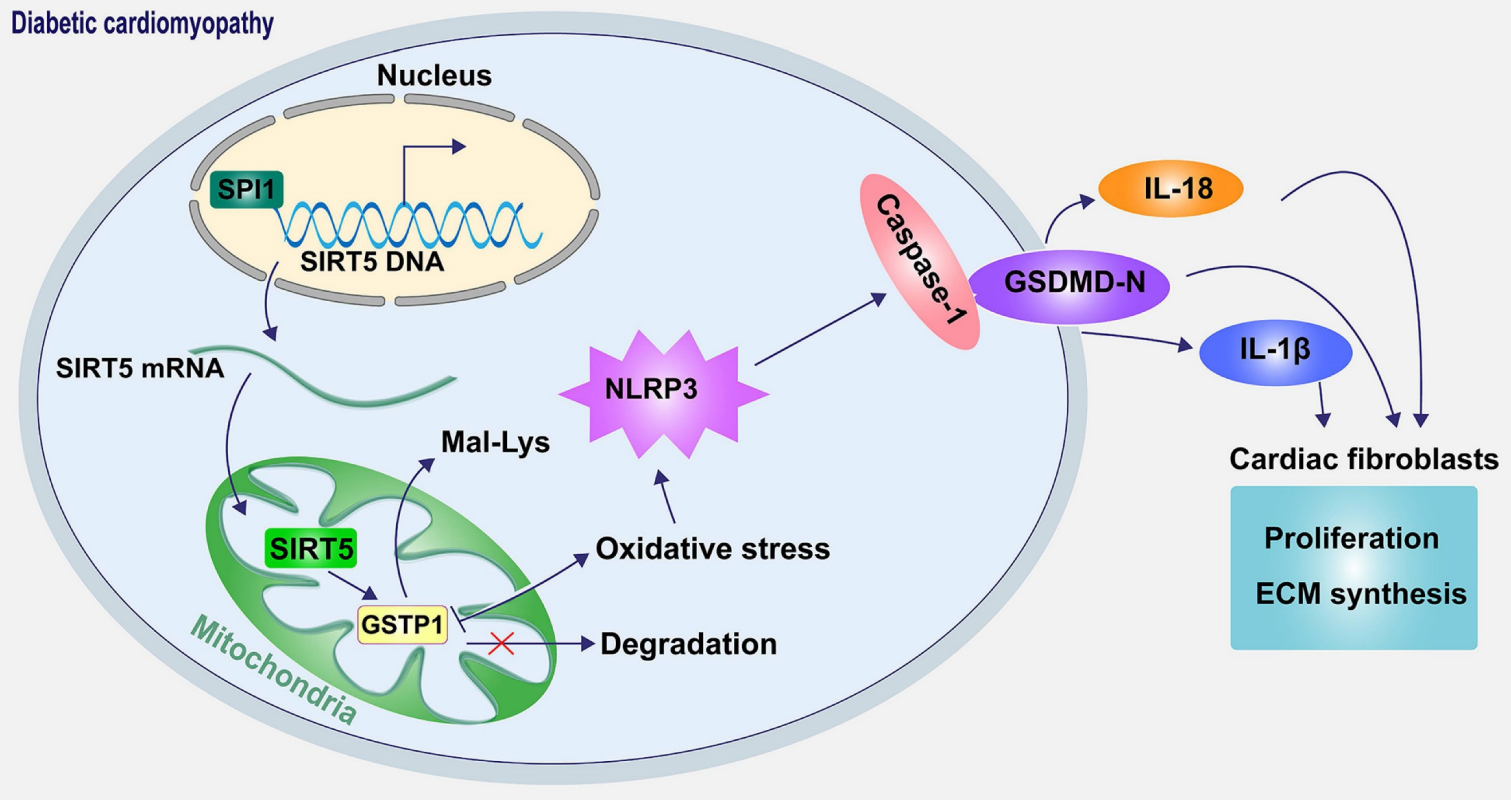
Schematic diagram of the mechanism. SPI1 inhibits oxidative stress-induced NLRP3 activation and subsequent cell pyroptosis and attenuates pyroptosis-induced cardiac fibroblast activation and ECM synthesis by promoting the transcription of SIRT5 and mediating the lysine demalonylation of GSTP1.

In conclusion, our results revealed a novel mechanism by which SIRT5-mediated lysine demalonylation of GSTP1 plays a protective role against oxidative stress and pyroptosis in DCM mice. Specifically, the pivotal role of SIRT5 appears critical in cardiac protection. Moreover, our results establish the downregulation of SIRT5 was caused by SPI1 deletion (Fig. 10). The basic knowledge reported in the present study provides a strategy that treatment with SIRT5 could be a promising pharmacological intervention for attenuating oxidative stress and pyroptosis in DCM.

## Supplementary Material

Supplementary figures.

## Figures and Tables

**Table 1:** Primers for the RT-qPCR

Gene	Forward primer (5'-3')	Reverse primer (5'-3')
SPI1	GAGGTGTCTGATGGAGAAGCTG	ACCCACCAGATGCTGTCCTTCA
SIRT5	ATCGCAAGGCTGGCACCAAGAA	CTAAAGCTGGGCAGATCGGACT
SIRT5 promoter	CCACCAATAGTATGTCGCGC	CGAAACAAAGCCCAGACCC
GSTP1	TGGAAGGAGGAGGTGGTTACCA	GGTAAAGGGTGAGGTCTCCATC
GAPDH	CATCACTGCCACCCAGAAGACTG	ATGCCAGTGAGCTTCCCGTTCAG

**Note:** SIRT5, sirtuin 5; GSTP1, glutathione S-transferase P; GAPDH, glyceraldehyde-3-phosphate dehydrogenase.

**Table 2:** Mal-Lys modification site of GSTP1

Position	Peptide	Posterior probability Score	CutOff
103	VEDLRG K YVTLIYTNY	0.9913	0.5
121	GKNDYV K ALPGHLKPF	0.924	0.5
128	ALPGHL K PFETLLSQN	0.9934	0.5
191	SARPKI K AFLSSPEHV	0.7417	0.5

**Note:** GSTP1, glutathione S-transferase P; Mal-Lys, lysine malonylation.
