# Supplementary material for: SIRT5-related lysine demalonylation of GSTP1 contributes to cardiomyocyte pyroptosis suppression in diabetic cardiomyopathy
Source: Int J Biol Sci. 2024 Jan 1;20(2):585–605. doi: 10.7150/ijbs.83306 (PMC10758093; doi:10.7150/ijbs.83306)
Supplement: Supplementary file 1 — Supplementary figures. [file ijbsv20p0585s1.pdf]

Supplementary Figure S1

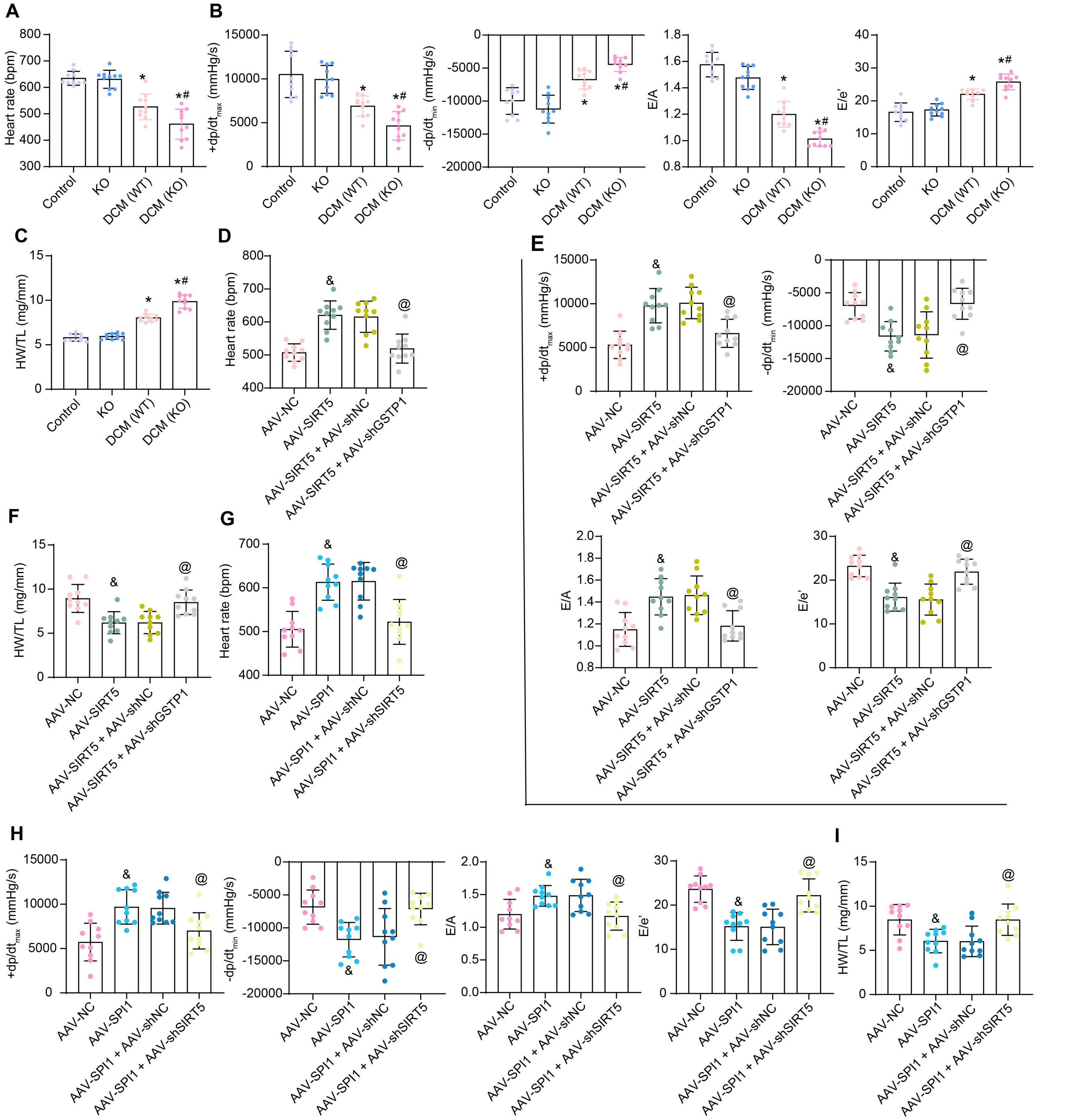

Supplementary Figure S1 Hemodynamics and diastolic function of mice with different treatments. (A) Heart rate in WT and SIRT5 KO mice under normal and DCM modeling conditions. (B) Hemodynamic data of WT and SIRT5 KO mice in normal and DCM modeling conditions. (C) HW/TL in WT and SIRT5 KO mice in normal and DCM modeling conditions. (D) Effects of AAV-SIRT5 and

AAV-shGSTP1 on heart rate in WT mice with DCM. (E) Effect of AAV-SIRT5 and AAV-shGSTP1 on hemodynamics in WT mice with DCM. (F) Effects of AAV-SIRT5 and AAV-shGSTP1 on HW/TL in WT mice with DCM. (G) Effects of AAV-SPI1 and AAV-shSIRT5 on heart rate in WT mice with DCM. (H) Effects of AAV-SPI1 and AAV-shSIRT5 on hemodynamics in WT mice with DCM. (I) Effects of AAV-SPI1 and AAV-shSIRT5 on HW/TL in WT mice with DCM. All data are expressed as means ± SD (n = 10). *$p < 0.05$ *vs.* control group; #$p < 0.05$ *vs.* DCM (WT) group; &$p < 0.05$ *vs.* AAV-NC group; @$p < 0.05$ *vs.* AAV-SPI1 + AAV-shNC or AAV-SIRT5 + AAV-shNC group. Continuous data were compared by one-way/two-way ANOVA.

Supplementary Figure S2

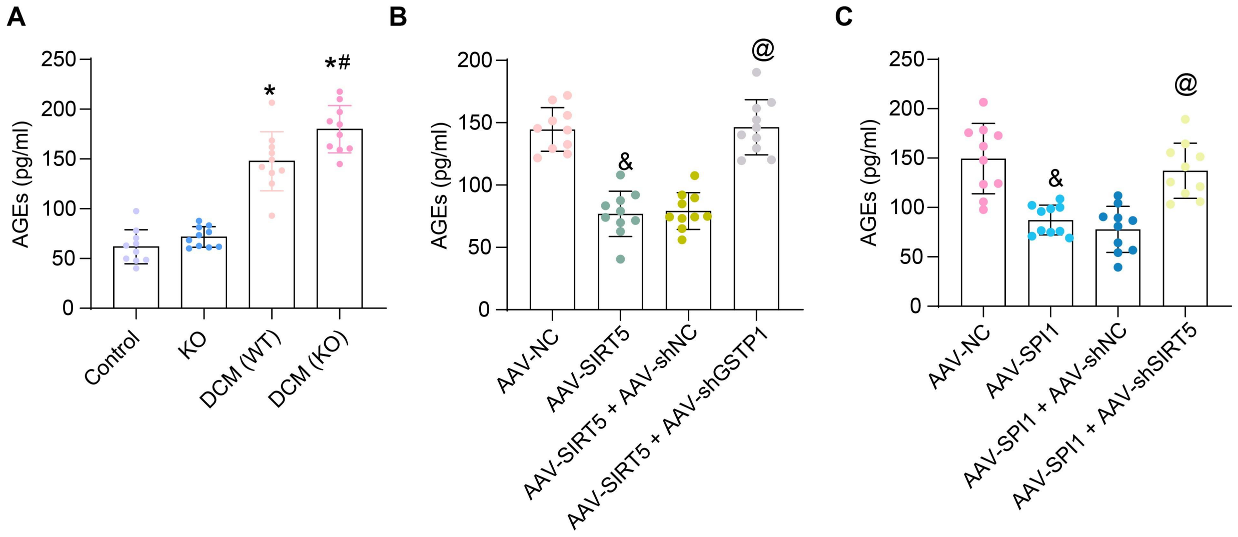

Supplementary Figure S2 Quantification of AGEs in mouse myocardial tissue. (A-C) The concentrations of AGEs in the myocardial tissues of mice in each group were measured by ELISA kits. All data are expressed as means ± SD (n = 10). *p < 0.05 *vs.* control group; #p < 0.05 *vs.* DCM (WT) group; &p < 0.05 *vs.* AAV-NC group; @p < 0.05 *vs.* AAV-SPI1 + AAV-shNC or AAV-SIRT5 + AAV-shNC group. Continuous data were compared by one-way ANOVA.

Supplementary Figure S3

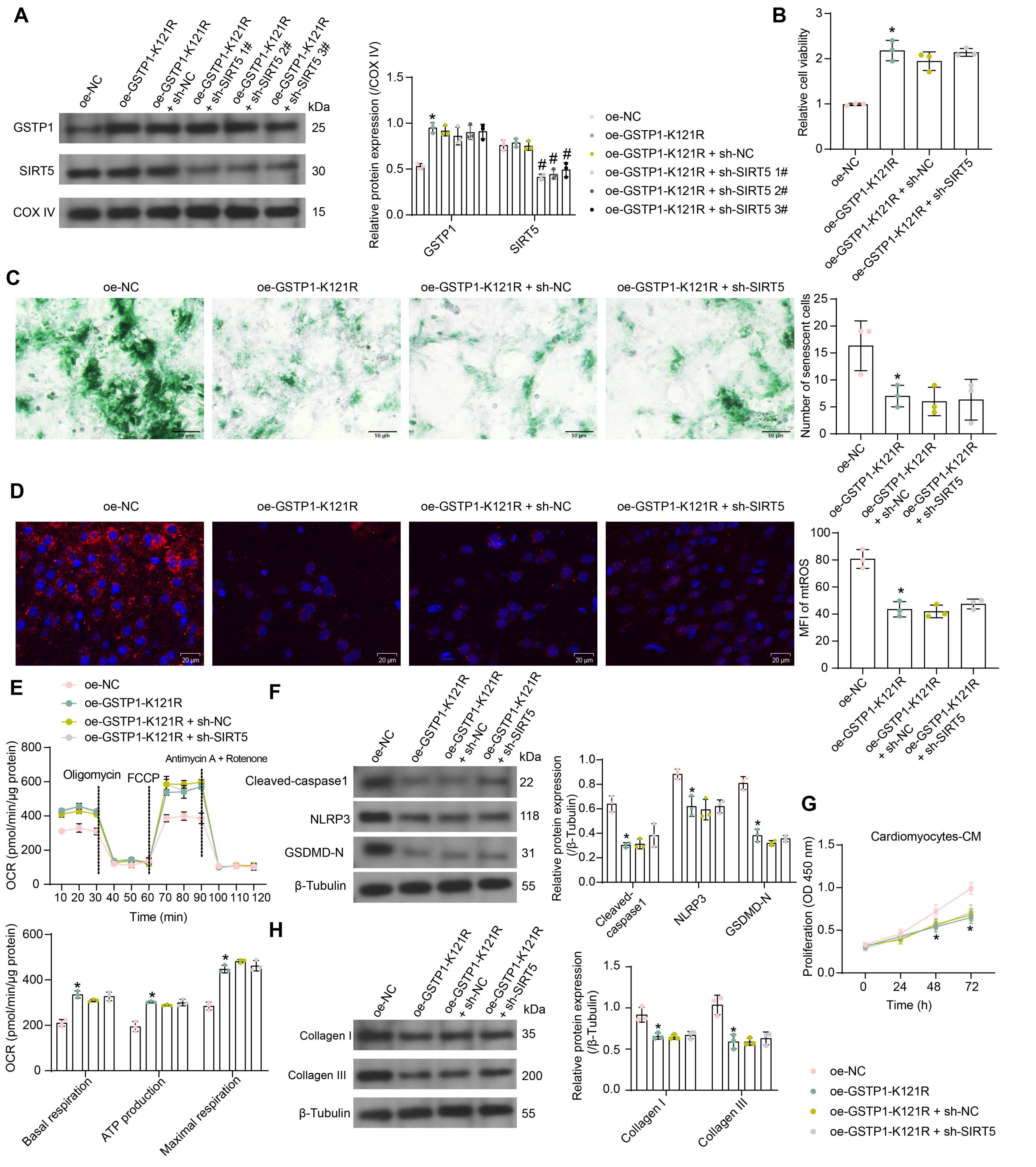

Supplementary Figure S3 SIRT5 shows an insignificant impact on the cardiomyocyte protective effect of GSTP1-K121R. (A) The effect of transfection of GSTP1-K121R plasmid and SIRT5 shRNAs on the expression of GSTP1 and SIRT5 in HG-treated cardiomyocytes was examined using Western

blot. (B) The viability of HG-treated cardiomyocytes was examined using CCK-8 assays. (C) HG-treated cardiomyocyte senescence was detected using β-galactosidase staining. (D) The mtROS production in HG-treated cardiomyocytes using MitoSox Red staining. (E) The mitochondrial function of HG-treated cardiomyocytes was assessed by seahorse assay. (F) The expression of pyroptosis-related proteins in HG-treated cardiomyocytes using Western blot. (G) Effect of the CM of cardiomyocytes after transfection and exposure to HG on the proliferative capacity of cardiac fibroblasts. (H) Effect of cardiomyocytes after transfection and exposure to HG on ECM synthesis in cardiac fibroblasts. All data are expressed as means ± SD (n = 3). *$p < 0.05$ *vs.* oe-NC group; #$p < 0.05$ *vs.* oe-GSTP1-K121R + sh-NC group. Continuous data were compared by one-way/two-way ANOVA.
